# Impact of Two Neuronal Sigma-1 Receptor Modulators, PRE084 and DMT, on Neurogenesis and Neuroinflammation in an Aβ_1–42_-Injected, Wild-Type Mouse Model of AD

**DOI:** 10.3390/ijms23052514

**Published:** 2022-02-24

**Authors:** Emőke Borbély, Viktória Varga, Titanilla Szögi, Ildikó Schuster, Zsolt Bozsó, Botond Penke, Lívia Fülöp

**Affiliations:** Department of Medical Chemistry, University of Szeged, Dóm Tér 8, H-6720 Szeged, Hungary; emokeborbely@gmail.com (E.B.); vargaviki666@gmail.com (V.V.); szogititi@gmail.com (T.S.); schuster.ildiko@med.u-szeged.hu (I.S.); bozso.zsolt@med.u-szeged.hu (Z.B.); penke.botond@med.u-szeged.hu (B.P.)

**Keywords:** Alzheimer’s disease, Aβ_1–42_-induce mouse model, neurogenesis, neuroinflammation, sigma-1 receptor, dimethyltryptamine, PRE084

## Abstract

Alzheimer’s disease (AD) is the most common form of dementia characterized by cognitive dysfunctions. Pharmacological interventions to slow the progression of AD are intensively studied. A potential direction targets neuronal sigma-1 receptors (S1Rs). S1R ligands are recognized as promising therapeutic agents that may alleviate symptom severity of AD, possibly via preventing amyloid-β-(Aβ-) induced neurotoxicity on the endoplasmic reticulum stress-associated pathways. Furthermore, S1Rs may also modulate adult neurogenesis, and the impairment of this process is reported to be associated with AD. We aimed to investigate the effects of two S1R agonists, dimethyltryptamine (DMT) and PRE084, in an Aβ-induced in vivo mouse model characterizing neurogenic and anti-neuroinflammatory symptoms of AD, and the modulatory effects of S1R agonists were analyzed by immunohistochemical methods and western blotting. DMT, binding moderately to S1R but with high affinity to 5-HT receptors, negatively influenced neurogenesis, possibly as a result of activating both receptors differently. In contrast, the highly selective S1R agonist PRE084 stimulated hippocampal cell proliferation and differentiation. Regarding neuroinflammation, DMT and PRE084 significantly reduced Aβ_1–42_-induced astrogliosis, but neither had remarkable effects on microglial activation. In summary, the highly selective S1R agonist PRE084 may be a promising therapeutic agent for AD. Further studies are required to clarify the multifaceted neurogenic and anti-neuroinflammatory roles of these agonists.

## 1. Introduction

Alzheimer’s disease (AD) is the most common form of dementia, characterized by progressive memory loss, impaired learning, and cognitive dysfunction. The main pathological hallmarks of AD are extracellular amyloid plaques and intracellular neurofibrillary tangles accumulated in the cerebral tissue [1], which first appear in the hippocampal and entorhinal regions of the brain, explaining the impairment of cognitive functions [2]. These changes are accompanied by the damage of synaptic connections, and neuronal death. The abnormal cleavage of amyloid precursor protein (APP) by β- and γ-secretases predominantly yields 40 to 43 amino acid long amyloid-β (Aβ) peptides, which aggregate, and manifest as cerebral deposits. Besides forming plaques, these oligomeric forms of Aβ are also thought to be neurotoxic [3,4,5,6]. These short oligomers might interfere with crucial intracellular mechanisms and signaling pathways. Thus, they may affect cell homeostasis, proliferation, differentiation, and survival [7,8,9,10]. Another significant symptom of AD is neuroinflammation, which involves various inflammatory components, such as immune cells, cytokines, and chemokines. Neuroinflammation might significantly alter neurogenesis, as well as enhancing Aβ production and plaque formation [11,12,13]. Currently, there is no cure for AD, and its progression cannot be prevented; at present, only symptomatic treatments of mild to moderate efficiency are available. Therefore, effective disease-modifying therapeutics that may halt the progression of AD and contribute to the protection of neuronal integrity are eagerly awaited. A potentially new direction of the research aiming to find novel disease-modulating agents targets the sigma receptors (SRs). SRs have received considerable attention for their potential role in the prevention of Aβ-induced neurotoxicity, as well as in the regulation of the pathophysiology of AD. Furthermore, SRs may be essential for modulating neurogenesis in adulthood, and the stimulation of this process has been linked to AD. Thus, SR ligands are being recognized as promising therapeutic agents for treating or alleviating AD [6,14,15,16].

Two subtypes of SRs are distinguished, sigma-1 receptor (S1R) and sigma-2 receptor [17,18,19]. S1R is broadly expressed in the central nervous system (CNS), especially in the dentate gyrus (DG) region of the hippocampus (HC), both in neurons and glial cells. S1Rs are mainly located in a specific part of the cell where the endoplasmic reticulum (ER) and the mitochondria establish a tight interplay; this area is called the mitochondria-associated ER membrane (MAM) [16,20,21,22,23]. S1R is known to influence neuronal survival, proliferation, neurite growth, plasticity, as well as learning and memory functions [24,25,26,27]. It has been reported that the expression level of S1R decreases in patients with neurodegenerative diseases like AD [16,22,23,28,29,30,31,32,33].

S1R binds a diverse set of molecules, for example, antipsychotics, antidepressants, and neurosteroids [34,35,36,37]. A non-specific endogenous ligand of S1R is N,N-dimethyltryptamine (DMT), a hallucinogenic agent assumed to be produced in small quantities and accumulated in the CNS [16,38,39,40]. Previous studies have shown that the administration of DMT modulates many ion channels [39], protects against hypoxia-induced damage [41], alleviates neuroinflammation [42,43], increases the density of dendritic spines [44], as well as promotes neurogenesis and neuritogenesis [45,46,47,48,49]. However, DMT might also exert anxiogenic, neuro- and cytotoxic effects [47,50,51,52]. DMT is known to bind to several receptors with different affinities: 5-hydroxytryptamine (5-HT)_1A-B,_ 5-HT_1D_, 5-HT_2A-C_, 5-HT_5A_, 5-HT_6_, 5-HT_7_ receptors, S1R, SERT, dopamine (D)_1-5_ receptors, α_1_AR, I_1-3_, TAAR, NMDA [53,54,55]. Several adverse effects of DMT are primarily associated with the stimulation of 5-HT_2A_ receptors [47,50,51,53,56], while its positive impacts are rather related to the activation of S1Rs [40,41,42,43,44,46,49,50,52,57]. Moreover, the inflammation regulatory and plasticity promoting activities of DMT are also considered to result from its binding to both the S1Rs and 5-HT receptors. Identifying the valid contributor molecules and signaling pathways behind this assumption requires more convincing evidence.

Many exogenous ligands of S1R have been identified, including (+)-pentazocine, fluvoxamine, ANAVEX2-73, and 2-(4-morpholinethyl)-1-phenylcyclohexanecarboxylate (PRE084) [16,22,24,58]. The antidepressant and nootropic properties of PRE084 are also recognized [59]. Based on our current knowledge, PRE084 may promote neuroprotection and neurite growth by stimulating the expression of different neurotrophic factors, as well as by activating signaling pathways involved in cell survival [60,61,62,63,64,65]. Previous studies suggest that this S1R-agonist might positively impact learning and memory, as demonstrated in animal models of neurodegenerative diseases or traumatic brain injuries [63,64,66]. It is also reported that after the administration of Aβ_25–35_ infusion into the right lateral ventricle of mice, PRE084 administration has moderated the adverse behavioral effects of Aβ_25–35_ [27] via reducing neurotoxicity-induced cell death [32,64]. Moreover, PRE084 may also promote neurogenesis [9] and cell survival by attenuating excitotoxicity and reducing microglial activity, as well as diminishing the expression of proinflammatory factors [67,68].

As mentioned above, in addition to its ability to support cell survival under stress conditions, activated S1Rs may also stimulate the formation of new neurons, even in the adult brain. In adulthood, mammalian neurogenesis is derived from neuronal stem cells (NSCs) located in the subgranular zone (SGZ) of the dentate gyrus (DG) in the hippocampus (HC), as well as from NSCs in the subventricular region of the lateral ventricles [69,70]. After differentiation and migration, these newly formed neurons can integrate into local neuronal circuits of the HC; thus, they might have a significant role in plasticity, cognitive functions, learning, and memory processes [71]. An optimal microenvironment is essential for the division, differentiation, migration, and maturation of NSCs. Physiologically, the activity of adult hippocampal neurogenesis decreases with aging, leading to a usually mild, age-associated cognitive decline. However, a growing body of evidence indicates that the extent of adult neurogenesis is sharply diminished in the early stages of AD, even before the appearance of senile plaques [72,73,74,75,76,77,78]. This finding raises the question of whether impaired neurogenesis may initiate and/or contribute to more severe cognitive deficits, thus mediating AD’s pathogenesis. Furthermore, these findings suggest that the stimulation of neurogenesis might serve as a therapeutic target in AD, with a potential to improve cognitive functions and promote neural adaptability, thereby it might prevent or even treat AD.

In this study, two main objectives were addressed. First, to induce early acute AD-like impairments in neurogenesis and generate neuroinflammation in adult wild-type C57BL/6 mice by the intracerebroventricular (ICV) administration of Aβ_1–42_ oligomers. In this experimental paradigm, we followed the administration protocol described by Li et al., who examined the effects of Aβ_25–35_ on the same processes [9]. They reported that Aβ_25–35_ stimulated the proliferation of neuronal progenitor cells, while enhancing the death of newly formed neurons and impaired neurite growth. Secondly, we attempted to restore the normal functioning of adult neurogenesis and reduce neuroinflammation by activating S1Rs with two different ligands, PRE084 and DMT. The intraperitoneally-(IP-)-injected compounds were tested in wild-type mice, either treated with Aβ_1–42_-oligomers or injected with vehicle (phosphate buffered saline (PBS)) as a control. Based on previously published articles on the beneficial effects of these S1R modulators, we expected to detect an obvious positive impact of the tested agents on the Aβ_1–42_-induced impairments in adult neurogenesis and neuroinflammation [41,42,43,49,52,57,60,61,62,63,64,79].

## 2. Results

### 2.1. Effects of PRE084 and DMT on Adult Neurogenesis in Aβ_1–42_ and Vehicle-Treated Mice

Aβ_1–42_ and DMT impair, while PRE084 promotes the survival of progenitor cells in DG.

**Figure 1:**
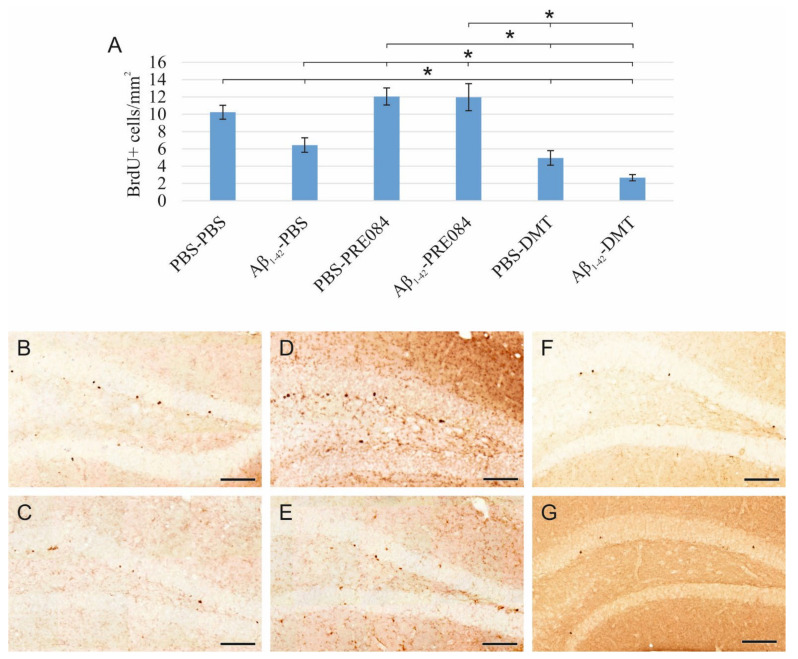
(**A**) Results for 5-Bromo-2′-Deoxyuridine (BrdU) immunolabeling. We observed significant differences in the quantity of stem cells between the six groups (ANOVA: *p* ≤ 0.0001). Significantly fewer BrdU+ cells were detected in the Aβ_1–42_-PBS, PBS-DMT, and in the Aβ_1–42_-DMT treated animals compared to the PBS-PBS group (PBS-PBS vs. Aβ_1–42_-PBS *p* = 0.001, vs. PBS-DMT *p* = 0.001, vs. Aβ_1–42_-DMT *p* ≤ 0.0001). The difference between the Aβ_1–42_-PBS and Aβ_1–42_-DMT treatment groups was also significant (*p* = 0.005). PRE084-treatment increased the number of stem cells detected in the SGZ; this change was significant in the Aβ_1–42_-administered group compared to its vehicle-treated control (Aβ_1–42_-PBS vs. Aβ_1–42_-PRE084 *p* ≤ 0.0001). The differences between the following groups in pairwise comparisons also reached significance: PBS-PRE084 vs. Aβ_1–42_-PBS *p* ≤ 0.0001, vs. PBS-DMT *p* ≤ 0.0001, vs. Aβ_1–42_-DMT *p* ≤ 0.0001; Aβ_1–42_-PRE084 vs. PBS-DMT *p* ≤ 0.0001, vs. Aβ_1–42_-DMT *p* ≤ 0.0001. (**B**–**G**) Representative images of BrdU staining: (**B**) PBS-PBS, (**C**) Aβ_1–42_-PBS, (**D**) PBS-PRE084, (**E**) Aβ_1–42_-PRE084, (**F**) PBS-DMT, (**G**) Aβ_1–42_-DMT. Scale bars represent 100 μm. *: *p* ≤ 0.05.

Proliferating cells were labeled by three IP injections of 5-Bromo-2′-Deoxyuridine (BrdU) with a 6 h interval, which was administered 24 h after the stereotaxic surgery. BrdU is a synthetic thymidine analog, which incorporates into the DNA strand, and can be detected by specific antibodies. We counted BrdU+ cells 14 days after the surgery. According to our results, the quantity of BrdU+ stem cells in the SGZ of the DG significantly differed among the six groups (ANOVA: *p* ≤ 0.0001). Aβ_1–42_ infusion significantly reduced the number of progenitor cells compared to the respective control group (PBS-PBS vs. Aβ_1–42_-PBS *p* = 0.001). Interestingly, significantly more severe negative changes were detected in animals treated with DMT. In those co-treated with both Aβ_1–42_ and DMT, hardly any BrdU+ stem cells were detected in the SGZ (Aβ_1–42_-DMT vs. PBS-PBS *p* ≤ 0.0001, vs. Aβ_1–42_-PBS *p* = 0.005, vs. Aβ_1–42_-PRE084 *p* ≤ 0.0001; PBS-DMT vs. PBS-PBS *p* = 0.001, vs. PBS-PRE084 *p* ≤ 0.0001). PRE084 treatment increased the amount of BrdU+ cells; the difference between the Aβ_1–42_-infused groups was significant (Aβ_1–42_-PBS vs. Aβ_1–42_-PRE084 *p* ≤ 0.0001) (Figure 1).

Aβ_1–42_ and PRE084 increase the number of premature cells, while DMT does not affect their quantity.

**Figure 2:**
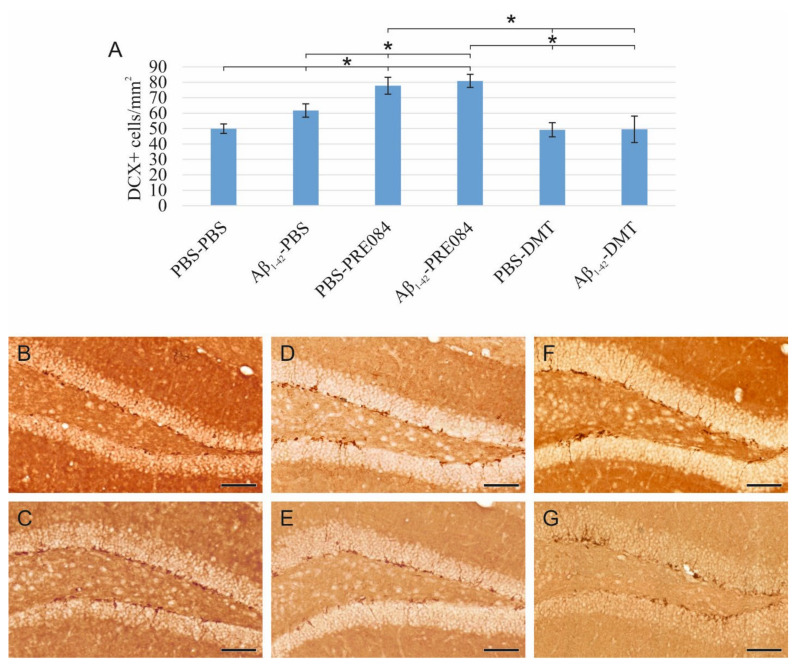
(**A**) Results for doublecortin (DCX) immunostaining. Detected DCX densities significantly differed among the six groups (ANOVA: *p* ≤ 0.0001). Compared to the control (PBS-PBS) animals, a significantly higher amount of DCX+ cells were detected in the Aβ_1–42_-PBS, PBS-PRE084 and Aβ_1–42_-PRE084-treated groups (PBS-PBS vs. Aβ_1–42_-PBS *p* = 0.037, vs. PBS-PRE084 *p* ≤ 0.0001, vs. Aβ_1–42_-PRE084 *p* ≤ 0.0001). Similarly, a significant difference was detected between the groups treated with Aβ_1–42_-PBS and Aβ_1–42_-PRE084 (*p* = 0.007). DMT treatment did not alter the number of immature neurons in the SGZ. Furthermore, significant differences were found when the groups were compared to the PBS-PRE084-treated group: PBS-PRE084 vs. Aβ_1–42_-PBS *p* = 0.023, vs. PBS-DMT *p* = 0.001, vs. Aβ_1–42_-DMT *p* = 0.001. Additional significant results were detected: Aβ_1–42_-PRE084 vs. PBS-DMT *p* ≤ 0.0001, vs. Aβ_1–42_-DMT *p* ≤ 0.0001. (B–G) Representative images of DCX immunolabeling: (**B**) PBS-PBS, (**C**) Aβ_1–42_-PBS, (**D**) PBS-PRE084, (**E**) Aβ_1–42_-PRE084, (**F**) PBS-DMT, (**G**) Aβ_1–42_-DMT. Scale bars represent 100 μm. *: *p* ≤ 0.05.

To understand the effects of PRE084 and DMT on the maturation of granule cells, we quantified immature neurons in the SGZ of DG. To label premature cells, we stained a microtubule-associated protein called doublecortin (DCX), which is expressed specifically in migrating neuronal precursors. The measured DCX densities were significantly different among the six groups (ANOVA: *p* ≤ 0.0001). In those treated with Aβ_1–42_-PBS and PBS-PRE084, the number of immature neurons was significantly higher compared to the control group (PBS-PBS vs. Aβ_1–42_-PBS *p* = 0.037, vs. PBS-PRE084 *p* ≤ 0.0001, vs. Aβ_1–42_-PRE084 *p* ≤ 0.0001). We also detected a significant difference between the Aβ_1–42_-PBS and Aβ_1–42_-PRE084 mice groups (*p* = 0.007). DMT administration did not affect the number of premature neurons compared to PBS-PBS mice (Figure 2).

The density of mature granule cells is unaffected by Aβ_1–42_ or PRE084 administration, while DMT induces a decrease in neuronal density.

**Figure 3:**
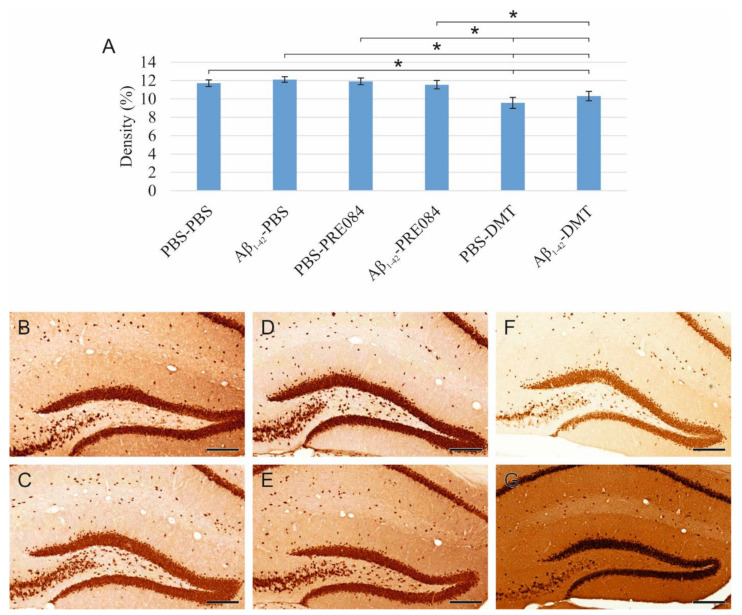
(**A**) Results for neuronal nuclei (NeuN) immunostaining. Significant differences were detected among the groups as follows (ANOVA: *p* = 0.001): in DMT-treated animals, significantly lower NeuN densities were evident compared to the PBS-PBS and Aβ_1–42_-PBS groups (PBS-DMT vs. PBS-PBS *p* = 0.001, vs. Aβ_1–42_-PBS *p* ≤ 0.0001; Aβ_1–42_-DMT vs. PBS-PBS *p* = 0.022, vs. Aβ_1–42_-PBS *p* = 0.003). Furthermore, significant differences were found when the groups were compared to the PBS-DMT-treated group: PBS-DMT vs. PBS-PRE084 *p* = 0.001, vs. Aβ_1–42_-PRE084 *p* = 0.006; Aβ_1–42_-DMT vs. PBS-PRE084 *p* = 0.024. (**B**–**G**) Representative photomicrographs of NeuN immunolabeling: (**B**) PBS-PBS, (**C**), Aβ_1–42_-PBS (**D**) PBS-PRE084, (**E**) Aβ_1–42_-PRE084, (**F**) PBS-DMT, (**G**) Aβ_1–42_-DMT. Scale bars represent 200 μm. *: *p* ≤ 0.05.

To detect and evaluate mature granule cells in the HC, we performed neuronal nuclei (NeuN) immunostaining (Figure 3). Again, significant differences were observed among the groups (ANOVA: *p* = 0.001). In DMT-treated animals, significantly lower NeuN+ cell densities were evident in the HC compared to the PBS-PBS and Aβ_1–42_-PBS group (PBS-PBS vs. PBS-DMT *p* = 0.001, vs. Aβ_1–42_-DMT *p* = 0.022; Aβ_1–42_-PBS vs. PBS-DMT *p* ≤ 0.0001, vs. Aβ_1–42_-DMT *p* = 0.003).

### 2.2. Effects of PRE084 and DMT on Neuroinflammation Induced by Aβ_1–42_

Aβ_1–42_ stimulates microglia activation, and neither PRE084, nor DMT alleviate this effect, while DMT alone significantly decreases microglial density.

**Figure 4:**
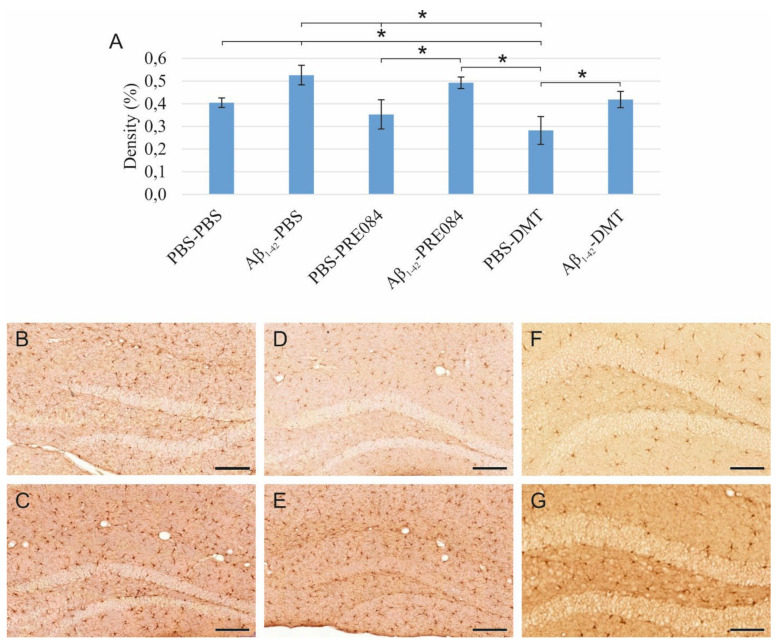
(**A**) Results for ionized calcium-binding adapter molecule 1 (Iba1) immunolabeling. Significant differences were observed among the groups (ANOVA: *p* = 0.002). Aβ_1–42_ increased the density of Iba1+ microglia significantly compared to PBS-PBS, PBS-PRE084, and PBS-DMT treated mice, respectively (PBS-PBS vs. Aβ_1–42_-PBS *p* = 0.015; PBS-PRE084 vs. Aβ_1–42_-PRE084 *p* = 0.035; PBS-DMT vs. Aβ_1–42_-DMT *p* = 0.039). The difference between the PBS-PBS and PBS-DMT groups was also significant (PBS-PBS vs. PBS-DMT *p* = 0.031). Moreover, significant differences were detected between the following groups: Aβ_1–42_-PBS vs. PBS-PRE084 *p* = 0.005, vs. PBS-DMT *p* ≤ 0.0001; Aβ_1–42_-PRE084 vs. PBS-DMT *p* = 0.002. (**B**–**G**) Representative images of Iba1 immunostaining: (**B**) PBS-PBS, (**C**) Aβ_1–42_-PBS, (**D**) PBS-PRE084, (**E**) Aβ_1–42_-PRE084, (**F**) PBS-DMT, (**G**) Aβ_1–42_-DMT. Scale bars represent 100 μm. *: *p* ≤ 0.05.

Neuroinflammation results from the activation of an immune response in the CNS, mediated by microglia and astrocytes. This process is induced by infective agents, neurodegenerative diseases, or injuries. To identify activated microglia in the HC, we stained ionized calcium-binding adapter molecule 1 (Iba1), expressed explicitly by monocyte-derived and resident macrophages, including microglia. Our results showed a significant difference in the density of Iba1+ microglia among the groups (ANOVA: *p* = 0.002). Aβ_1–42_ administration significantly increased the density of activated microglia compared to the vehicle-treated control groups (PBS-PBS vs. Aβ_1–42_-PBS *p* = 0.015; PBS-PRE084 vs. Aβ_1–42_-PRE084 *p* = 0.035; PBS-DMT vs. Aβ_1–42_-DMT *p* = 0.039). In the PBS-DMT group, the density of Iba1+ microglia was significantly reduced compared to PBS-PBS-treated animals (PBS-PBS vs. PBS-DMT *p* = 0.031). Still, none of the treatments were found to be able to alleviate the proinflammatory effect of Aβ_1–42_ (Figure 4).

Aβ_1–42_ stimulates astrocyte reactivation, while the administration of DMT or PRE084 reduces this effect.

**Figure 5:**
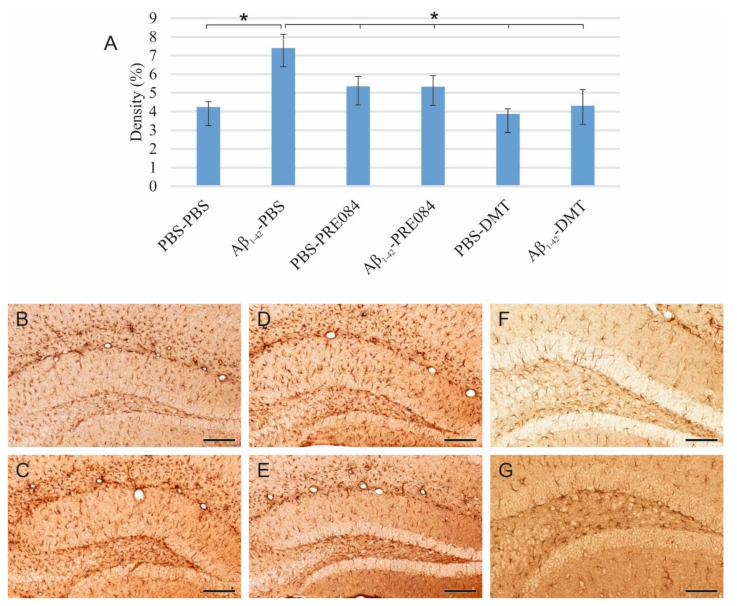
(**A**) Results of glial fibrillary acidic protein (GFAP) immunostaining. The densities of GFAP+ astrocytes differed among the groups (ANOVA: *p* ≤ 0.0001). A significantly higher GFAP+ density was detected in the Aβ_1–42_-PBS group compared to those treated with PBS-PBS (*p* ≤ 0.0001), PBS-PRE084 (*p* = 0.013), Aβ_1–42_-PRE084 (*p* = 0.013), PBS-DMT (*p* ≤ 0.0001), and Aβ_1–42_-DMT (*p* = 0.001). (**B**–**G**) Representative images of GFAP immunolabeling: (**B**) PBS-PBS, (**C**) Aβ_1–42_-PBS, (**D**) PBS-PRE084, (**E**) Aβ_1–42_-PRE084, (**F**) PBS-DMT, (**G**) Aβ_1–42_-DMT. Scale bars represent 100 μm. *: *p* ≤ 0.05.

Reactive astrocytes were immunostained for glial fibrillary acidic protein (GFAP), an intermediate filament protein expressed by different cell types, mainly reactive astrocytes, in the CNS. Significantly different GFAP+ cell densities were detected in the HC of the different groups (ANOVA: *p* = 0.002). A significant increase in the rate of reactivated astrocytes was detected in the Aβ_1–42_-PBS group compared to PBS-PBS-treated mice (*p* ≤ 0.0001). Furthermore, GFAP+ cell densities were significantly lower in all other groups compared to Aβ_1–42_-PBS-treated mice (Aβ_1–42_-PBS vs. PBS-PRE084 *p* = 0.013, vs. Aβ_1–42_-PRE084 *p* = 0.013, vs. PBS-DMT *p* ≤ 0.0001, vs. Aβ_1–42_-DMT, *p* = 0.001). The stimulatory effect of Aβ_1–42_ on astrocyte reactivation was alleviated by PRE084 and DMT administration (Figure 5).

The activation of inflammatory processes was assessed by the determination of certain proinflammatory cytokines (IL1β and TNFα). The levels of both pro- IL1β and soluble IL1β, as well as membrane-bound TNFα and soluble TNFα, were determined by western blot analyses (see Supplement Figure S1). These results corroborate our findings regarding the activation of the glial immunodefense system in response to the Aβ_1–42_ stimulus. The production of the active cytokine forms could be modulated by DMT-treatment; however, only the change in TNFα-level was significant.

### 2.3. S1R Protein Level Is Elevated by Aβ_1–42_ Treatment, as Well as by the Co-Administration of Aβ_1–42_ and PRE084 or DMT

**Figure 6:**
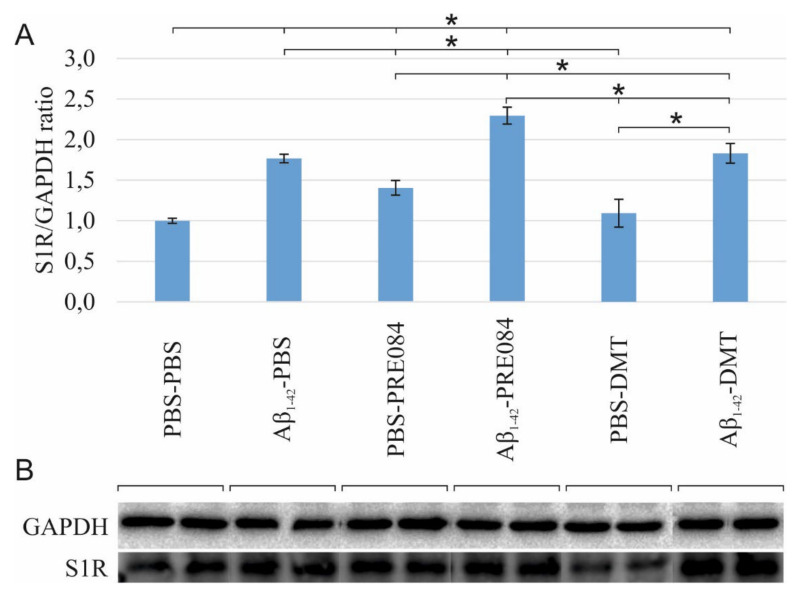
(**A**) Results for the western blot (WB) analysis. Significant differences were observed in the S1R levels among the groups (ANOVA: *p* ≤ 0.0001). Compared to PBS-PBS-treated mice, the S1R protein levels were significantly elevated in the Aβ_1–42_-PBS (*p* ≤ 0.0001), PBS-PRE084 (*p* = 0.018), Aβ_1–42_-PRE084 (*p* ≤ 0.0001), and Aβ_1–42_-DMT (*p* ≤ 0.0001) groups. In PBS-DMT-treated mice, the S1R protein expression remained close to the control level (*p* = 0.540), while S1R levels were somewhat higher in the PRE084-treated groups (PBS-PBS vs. PBS-PRE084 *p* = 0.018; Aβ_1–42_-PBS vs. Aβ_1–42_-PRE084 *p* = 0.004; PBS-PRE084 vs. Aβ_1–42_-PRE084 *p* ≤ 0.0001). In contrast, the co-administration of Aβ_1–42_ and DMT induced a significant increase in the quantity of S1R (PBS-DMT vs. Aβ_1–42_-DMT *p* ≤ 0.0001). Furthermore, significant differences were detected in the S1R expression upon the pairwise comparisons of the following groups: Aβ_1–42_-PBS vs. PBS-PRE084 *p* = 0.032, vs. PBS-DMT *p* = 0.001; Aβ_1–42_-PRE084 vs. PBS-DMT *p* ≤ 0.0001, vs. Aβ_1–42_-DMT *p* ≤ 0.0001, respectively. (**B**) WB gel electrophoresis images of S1R and GAPDH lines of the experimental groups. *: *p* ≤ 0.05.

To determine the effects of Aβ_1–42_ and PRE084 or DMT on the expression of S1R, a western blot (WB) analysis using GAPDH loading control was performed on HC and cerebral cortex samples of three animals per group. Our findings revealed a significant difference in the S1R levels among the groups (ANOVA: *p* ≤ 0.0001). S1R protein levels were significantly elevated in all groups, except in PBS-DMT-treated animals, as compared to control subjects (PBS-PBS vs. Aβ_1–42_-PBS *p* ≤ 0.0001, vs. PBS-PRE084 *p* = 0.018, vs. Aβ_1–42_-PRE084 *p* ≤ 0.0001, vs. PBS-DMT *p* = 0.540; vs. Aβ_1–42_-DMT *p* ≤ 0.0001, respectively). In comparison with Aβ_1–42_-PBS-treated mice, the Aβ_1–42_-PRE084 (*p* = 0.004) and Aβ_1–42_-DMT (*p* = 0.673) groups showed higher protein levels, while significantly lower levels of S1R were detected in PBS-PRE084 (*p* = 0.032) and PBS-DMT (*p* = 0.001) treated mice. As expected, the co-administration of Aβ_1–42_ and either of the S1R agonists increased the S1R protein level compared to the respective control group (Aβ_1–42_-PRE084 vs. PBS-PRE084 *p* ≤ 0.0001; Aβ_1–42_-DMT vs. PBS-DMT *p* = 0.015). Notably, the expression of S1R was significantly increased in Aβ_1–42_-PRE084-treated animals compared to the Aβ_1–42_-DMT group (*p* ≤ 0.0001). (Figure 6).

## 3. Discussion

During neurogenesis in adulthood, new neurons continuously develop and differentiate from hippocampal stem cells, and are integrated into existing neuronal networks to maintain plasticity of the CNS, and thereby preserve learning and memory functions. It has been recognized that the formation of new neurons reduces with age, manifesting in impaired cognitive functions [80]. In certain neurodegenerative diseases this cluster of mental symptoms is much more pronounced due to a decreased rate of neurogenesis, increased destruction of mature neurons, and enhanced neuroinflammatory responses. The most prevalent disease of this kind is AD, characterized by progressive dementia. Early alternations in adult neurogenesis and neuroinflammation may appear several years or even a decade before the diagnosis of AD, and probably contributes to the onset of neurological symptoms. It is hypothesized that an intensive stimulation of hippocampal neurogenesis and the reduction in neuroinflammation in adulthood could slow down the rate of decline of cognitive skills. Moreover, the uniquely structured S1R protein, functioning as a ligand-operated chaperone, is known to play a major role in both neurogenesis and neuroinflammation. Thus, it is assumed that the activation of S1Rs may be a promising therapeutic strategy to stimulate adult neurogenesis and alleviate neuroinflammatory processes.

The first objective of our study was to model these early alternations appearing in AD. Our experimental paradigm was based on the work of Li et al., in a modified way: instead of Aβ_25–35_, we injected Aβ_1–42_ ICV to induce early AD-like changes [9]. The reason for this modification is that Aβ_25–35_ is a non-natural, truncated sequence, and although it is prone to aggregation, its kinetics for aggregation differ from that of the native Aβ_1–42_ peptide. Therefore, using this latter peptide should yield biologically more relevant findings [81]. In the work of Li et al., neurogenesis was assessed 14 and 28 days after the peptide injections, and significant differences were detected on day 28 in neurogenic markers compared to baseline (reduced proliferation and neurite growth, increased death of newly formed cells) [9]. In our experimental model, AD-like cerebral neurogenic and neuroinflammatory changes could be detected as early as two weeks after the administration of Aβ_1–42_. We demonstrated that a single administration of Aβ_1–42_, directly into the lateral ventricles, significantly impaired the proliferation and increased the number of immature cells in mice. The effects of Aβ on neurogenesis are highly controversial in the literature. Numerous reports indicate that Aβ significantly decreases the formation of new neurons, possibly by impairing their ability to divide, as well as by diminishing the survival of neuronal stem cells in DG [7,8,9,75,76,77,82]. However, some research groups have published that Aβ can induce the initial proliferation step of neuron formation in different transgenic mouse strains [9,78,83,84,85] or in cellular models of AD [86,87,88,89,90,91]. In our experiments, an increase in the number of differentiating immature neurons was observed in Aβ_1–42_-treated animals, which may be explained by a compensatory cerebral mechanism [77,92]. Specifically, this enhancement of neuronal cell differentiation may be a response to the disturbed homeostasis resulting from the decrease in the stem cell population, aiming to restore the balance within the CNS. As we expected, in our experimental model, no significant reduction was detected in the density of mature, functional neurons in HC two weeks after the administration of Aβ_1–42_, indicating that the existing neuronal system may remain unaffected. Regarding neuroinflammation, we found that a single administration of Aβ_1–42_ stimulated neuroinflammatory processes, causing a significant increase in the densities of activated microglia and hyperreactive astrocytes. In line with our observations, several in vivo experiments have demonstrated the neuroinflammation-inducing effects of Aβ fibrils and oligomers injected into the brain tissue in different experimental models [93,94,95]. This neuroinflammatory environment may affect adult neurogenesis either positively or negatively [11,12,96,97,98,99,100,101]. It is known that cytokines and chemokines produced by activated microglia and astrocytes play an important role in neuroinflammatory processes. Certain anti-(IL-4, IL-10) and proinflammatory (IL-6, TNF-α) factors substantially influence neurogenesis, e.g., they can diminish proliferation and cell survival, while they may also stimulate cell differentiation [13]. Thus, beyond its direct effects on immature neurons, Aβ_1–42_ may also affect neurogenesis by generating a relatively mild, but chronic neuroinflammatory environment. Further research is needed to clarify the relative contribution of these two processes (direct and indirect) to the final decline of adult neurogenesis in AD.

Since the S1R protein plays a major role in neurogenesis and neuroinflammation, and changes in S1R expression levels have not been studied in exogenous Aβ-induced AD models, we examined the expression levels of this protein. In our case, the expression of S1R increased after a single administration of Aβ_1–42_. This finding may contradict some literature data, which report on the down-regulation of S1R in the early stage of human AD [24]. In the reported cases, both the amount and the binding potential of S1R were found to be decreased, presumably as a consequence of hippocampal neuronal death [24,102,103,104,105]. In contrast, other studies indicate that AD-related ER-stress can lead to an up-regulation of S1R [16,29,106,107], which, serving as a chaperon, modulates the canonical unfolded protein response (UPR) pathways (PERK, IRE1a, ATF6) [16,108]. In our study, the observed elevation of the level of S1R may be a consequence of the cytotoxic effect of Aβ_1–42_, which induces ER stress, and thus activates the UPR pathways and upregulates S1R expression. 

To date, the biological effects of DMT and PRE084 have not been studied in an Aβ-induced model of early AD with demonstrated changes in neurogenesis and S1R expression levels, as well as neuroinflammation. Therefore, we aimed to assess whether the modulation of S1Rs with selected ligands can restore Aβ_1–42_-induced alternations in adult neurogenesis and reduce neuroinflammation.

In our study, DMT significantly reduced the number of neuronal stem cells and densities of neurons. Similar to this finding, another tryptamine, psilocybin (4-phosphoryloxy-N, N-dimethyltryptamine) with a chemical structure close to that of DMT and a high binding affinity to 5-HT_2A_ receptors (Kd = 6 nM), was also found to impair synaptic growth and neurogenesis (proliferation and neuronal survival) [109]. However, the neuroprotective and neurogenesis stimulating effects of DMT and its analog, 5-methoxy-DMT, exerted via S1Rs, were also described in in vitro cell cultures and in a wild-type rodent model [44,46,49,54]. In our study, DMT was administered at a concentration of 1 mg kg^–1^, thus it is supposed to have occupied both receptor types, so their mixed effects could have been observed. Comparison of the Kd values (DMT-S1R Kd = 14.75 μM, DMT-5-HT_2A_ receptor Kd = 130 nM) indicates that DMT binds to the 5-HT_2A_ receptor with higher affinity than to S1R; thus, it is more likely to act on the 5-HT_2A_ receptors than on S1R [39,53]. Therefore, we suppose that DMT exerted its negative effect on neurogenesis via the 5-HT_2A_ receptors. The results of our WB analysis support this hypothesis, since the expression of the S1R protein was only slightly elevated after DMT treatment. 

Regarding the relation of DMT and neuroinflammation, conflicting findings are published in the literature. Some of them support the theory that DMT can alleviate neuroinflammatory processes, thus it may reduce the density of reactive astrocytes [41,42,43,52,57]. This effect may be related to the ability of DMT to bind to S1R [41,42,43,52], but the serotonergic receptors may also have roles in this process [110]. Morales-Garcia et al. reported that DMT induces a significant increase in the density of GFAP+ astrocytes via the activation of S1Rs, but these researchers conclude that this elevated GFAP level promotes neurogenesis [49]. In our experiments, DMT treatment was found to exert a positive effect on activated microglia and hyperreactive astrocytes against the Aβ_1–42_-induced neurotoxicity, but it was not detected to promote neurogenesis.

These contradictory results may be explained by the application of different protocols (injection and doses of BrdU and DMT, different survival times). It is also known that although DMT can penetrate the blood-brain barrier, upon exogenous administration its concentration in the CNS is elevated for a relatively short time only (elimination half-life ~15 min [44]). Therefore, it is also possible that in our model, the concentration of DMT in the CNS after IP administration was not sufficient to exert its effects on S1R as Morales-Garcia reported [49]. Further experiments are required to elucidate the exact mode of action of DMT regarding neurogenesis and neuroinflammation. 

To study the effect of an exogenous S1R agonist on neurogenesis and neuroinflammation, we applied PRE084 (Kd = 2.2 nM, [111]). Similarly, as Li et al. reported in an Aβ_25–35_-induced mouse model of AD, we have demonstrated that PRE084 promotes neurogenesis upon treatment with Aβ_1–42_, as it is indicated by the quantitative increase in stem cells and immature neurons after PRE084 administration. Furthermore, PRE084 per se activates cell proliferation, possibly by stimulating S1R.

Regarding neuroinflammation, the density of hyperreactive astrocytes and the degree of Aβ_1–42_-induced astrogliosis were reduced by the administration of PRE084. However, the substance neither per se, nor in combination with Aβ_1–42_ could impair microglial activation. It is known that in case of CNS tissue damage, activated microglia may behave either neurotoxic or neuroprotective, depending on their morphological and functional states. According to the literature, PRE084 can stimulate the proliferation of the anti-inflammatory type of microglia (M2), while it suppresses pro-inflammatory M1 microglia, thus it maintains the delicate balance between functional restorative and inflammatory glial phenotypes [62,112]. As we did not analyze the distribution and morphology of the microglia, we assume that the apparent ineffectiveness of PRE084 treatment on microglial activation may result from the above mentioned two mutual processes.

PRE084 binds to S1R with high affinity, either alone (compared to PBS and DMT controls) and when co-administered with Aβ_1–42_ (compared to Aβ_1–42_-PBS or Aβ_1–42_-DMT animals), and significantly induces the expression of this receptor protein. These results may confirm that PRE084 activates the S1R receptors effectively, so its neurogenic impact is more pronounced than that of DMT.

## 4. Materials and Methods

### 4.1. Animals

Male C57BL/6 wild-type mice (*n* = 80) from in-house breeding, weighing 23–28 g and aged 12 weeks at the beginning of the study, were used for the experiments. All animals, divided into groups, were kept under constant circumstances, including constant temperature (23 ± 0.5 °C), lighting (12:12 h light/dark cycle, lights on at 7 a.m.), and humidity (~50%). Standard mouse chow and tap water were supplied ad libitum. All behavioral experiments were performed in the light period. Handling was executed daily, at the same time, started one week before the experiments. All efforts were made to minimize the number of animals used, and their suffering throughout the experiments.

All experiments were performed in accordance with the European Communities Council Directive of 22 September 2010 (2010/63/EU on protecting animals used for scientific purposes). The experimental protocols were approved by the National Food Chain Safety and Animal Health Directorate of Csongrad County, Hungary (project license: XXVI./3644/2017). Formal approval to conduct the experiments was obtained from the Animal Welfare Committee of the University of Szeged (project No. I-74-16/2017, 04.07.2017).

### 4.2. Preparation and Structure Analysis of Aβ_1–42_ Peptide Oligomers

The iso-Aβ_1–42_ peptide was synthesized in the solid phase using tert-butyloxycarbonyl (Boc)-chemistry in-house, as reported earlier [113]. A stock solution of this peptide was prepared using distilled water, to yield a concentration of 1 mg/mL (200 μM, pH = 7), and it was sonicated for 3 min. The solution was incubated for 10 min at room temperature (RT), then the pH level was adjusted (pH = 11), and it was further incubated for 2 h. After a 3-min-long sonication process, the Aβ_1–42_ solution was diluted in phosphate buffer (PBS, 20 mM) to a final peptide concentration of 50 μM (26.67 mM phosphate, 1.2% NaCl, pH = 7.4). The solution was stored at 4 °C until further use on the same day.

The oligomeric state of the Aβ peptide was verified by a transmission electron microscope (JEM-1400, JEOL USA Inc., Peabody, MA, USA) operating at 120 kV. Images were taken by an EM-15300SXV system, routinely at a magnification of 25,000 and 50,000, and were processed by the SightX Viewer Software (EM-15300SXV Image Edit Software, JEOL Ltd., Tokyo, Japan).

### 4.3. Surgery, Solutions, and Drug Administration

Mice were anesthetized by an IP injection of a mixture of ketamine (10.0 mg/0.1 kg) and xylazine (0.8 mg/0.1 kg). The animals were then placed into a stereotaxic apparatus (David Kopf Instruments, Tujunga, CA, USA; Stoelting Co., Wood Dale, IL, USA), a midline incision of the scalp was made, the skin and muscles were carefully retracted to expose the skull, and a hole was drilled above the target area. A single intracerebroventricular injection of either Aβ_1–42_ (50 μM) or PBS (20 mM) was administered at the right side using a Hamilton syringe (32 G), injected at a rate of 0.5 μL/min. The following coordinates were used (from Bregma point): AP: −0.3; ML: −1.0; DV: −2.5. All animals were treated with antibiotics and analgesics after the surgery.

To detect stem cells, the animals were injected IP with BrdU (50 mg kg^–1^; Sigma-Aldrich, Saint Louis, MO, USA) dissolved in physiological saline, 3 times, 24 h after the surgery as described previously by Li et al. [9].

PRE084 (1 mg kg^–1^, Sigma-Aldrich, Saint Louis, MO, USA) and DMT (1 mg kg^–1^, Lipomed AG, Arlesheim, Switzerland) were also administered IP on a daily basis between postsurgery days 7–12. Both substances were dissolved in PBS (sterile-filtered, 20 mM) complemented with 1% dimethyl sulfoxide (DMSO, Sigma-Aldrich, Saint Louis, MO, USA).

Six groups of animals (with 18 mice in the control group, whereas 11 mice per group in the other groups) were developed to represent a control for each of the Aβ_1–42_-treated groups (i.e., those PBS-treated after the development of AD-like symptoms of impaired neurogenesis and neuroinflammation and those treated with DMT or PRE084 after the induction of neurogenic and neuroinflammatory changes). In the nomenclature of the groups, the first term refers the ICV administered solution (PBS or Aβ_1–42_), while the second one indicates the IP injected agent with potential disease-modifying activity (PBS again as a control, or PRE084 or DMT). Based on this nomenclature, the six groups were the following:

ICV: PBS-IP: PBS (PBS-PBS, i.e., PBS-treated, non-diseased control; n = 18),
ICV: Aβ_1–42_-IP: PBS (Aβ_1–42_-PBS, i.e., Aβ_1–42_-treated, PBS-treated control; n = 18),
ICV: PBS-IP: PRE084 (PBS-PRE084, i.e., PRE084-treated, non-diseased control; n = 11),
ICV: Aβ_1–42_-IP: PRE084 (Aβ_1–42_-PRE084, i.e., Aβ_1–42_-treated, PRE084-treated group; n = 11),
ICV: PBS-IP: DMT (PBS-DMT, i.e., DMT-treated, non-diseased control; n = 11),
ICV: Aβ_1–42_-IP: DMT (Aβ_1–42_-DMT, i.e., Aβ_1–42_-treated, DMT-treated group; n = 11).

### 4.4. Immunohistochemistry

Two weeks after the surgery, mice (n = 8-8 from the PRE084- and DMT-treated, and n = 15-15 from the control groups) were anesthetized with chloral hydrate (1 mg kg^–1^) and were perfused transcardially with PBS, followed by 4% paraformaldehyde (PFA, Sigma-Aldrich, St. Louis, MO, USA). All procedures after perfusion, including the post-fixation and the preparation of the slides, were executed the same way as described previously [114].

Immunohistochemical analysis was carried out on 20 µM formalin fixed cryosections. All immunohistochemical procedures were performed according to Szogi et al. [114]. All chemicals used in the immunohistochemical procedures, except the antibodies (Ab), were purchased from Sigma-Aldrich (St. Louis, MO, USA). Briefly, for BrdU staining, the sections were incubated in 2 M HCl for 2 h at RT to denature DNA. For the evaluation of BrdU-stained and NeuN-positive cells, the sections were blocked in a mixture of 8% normal goat serum, 0.3% bovine serum albumin (BSA), and 0.3% Triton X-100 in PBS for 1 h at RT. For DCX, Iba1 and GFAP labeling, the sections were blocked in a mixture of 0.1% BSA and 0.3% Triton X-100 in PBS for 1 h at RT. After this step, the slices were incubated at 4 °C overnight with primary antibodies added to the samples in the following dilutions: mouse anti-BrdU Ab (1:800; Santa Cruz Biotechnology, Dallas, TX, USA), goat anti-DCX Ab (1:4000; Santa Cruz Biotechnology, Dallas, TX, USA), mouse anti-NeuN Ab (1:500; Merck Millipore, Darmstadt, Germany), rabbit anti-Iba1 Ab (1:3600; Wako Chemicals GmbH, Neuss, Germany), and mouse anti-GFAP Ab (1:1500; Santa Cruz Biotechnology, Dallas, TX, USA). For BrdU, DCX, and NeuN stainings, the sections were treated with a polymer-based HRP-amplifying system (Super Sensitive^TM^ One-Step Polymer-HRP Detection System, BioGenex, Fremont, CA, USA), according to the manufacturer’s instructions. For Iba1 and GFAP labeling, the slices were incubated with the corresponding secondary antibodies: biotinylated goat anti-rabbit Ab (1:400; Jackson ImmunoResearch, West Grove, PA, USA), and biotinylated goat anti-mouse Ab (1:400; ThermoFisher Scientific, Waltham, MA, USA) for 60 min. Next, the sections were rinsed 3 times in PBS, and were incubated with avidin-biotin-complex (ABC Elite Kit; Vector Laboratories, Burlingame, CA, USA) for Iba1 in 1:1000 and for GFAP stainings in 1:1500, for 60 min at RT. The peroxidase immunolabeling was developed in 0.5 M Tris-HCl buffer (pH 7.7) with 3,3′-diaminobenzidine (10 mM) at RT in 30 min. The sections were mounted with dibutyl phthalate xylene onto the slides and were coverslipped.

### 4.5. Quantification of the Immunohistochemical Data

Slides were scanned by a digital slide scanner (Mirax Midi, 3DHistech Ltd., Budapest, Hungary), equipped with a Panoramic Viewer 1.15.4, a CaseViewer 2.1 program and a QuantCenter, HistoQuant module (3DHistech Ltd., Budapest, Hungary). For quantifications, all sections derived from each animal were analyzed. In DG and HC, the regions of interest (ROI) were manually outlined. Antibody-positive cell types were counted and quantified from ROIs. The number of stem cells (BrdU+) and neuroblasts (DCX+) were assessed by the observers. The densities (%) of neurons (NeuN+), microglia (Iba1+), and astrocytes (GFAP+) were calculated by the quantification software. To assess cell densities, we divided the total number of counted cells per animal with the DG/HC area, and represented them as cells/mm^2^ (BrdU+, DCX+) or % (NeuN+, Iba+, GFAP+).

### 4.6. Western Blot Analysis

To determine the effects of Aβ_1–42_ and PRE084 or DMT on the expression of S1R, the receptor protein samples of 3 animals per group (n = 18) were identically prepared, separated, and transferred to nitrocellulose membranes. The membranes were washed and treated as described by Szogi et al. [54]. The levels of S1R (mouse S1R antibody, Santa Cruz, Dallas, TX, USA, 1:1000) were analyzed in each group. For the analysis, we used glyceraldehyde 3-phosphate dehydrogenase (GAPDH, rabbit GAPDH antibody, Cell Signaling, Danvers, MA, USA, 1:200,000) as the loading control.

### 4.7. Statistical Analysis

The data obtained from the immunohistochemistry analyses were evaluated with a one-way ANOVA, followed by Fisher’s LSD post hoc tests. The WB data did not follow normal distribution; thus, they were analyzed with Kruskal-Wallis nonparametric tests, followed by Mann–Whitney U tests for multiple comparisons. Data were analyzed with the SPSS software (IBM SPSS Statistics 24), and the results were expressed as mean ± (SEM). Statistical significance was set at *p* ≤ 0.05.

## 5. Conclusions

Adult neurogenesis is essential for CNS plasticity. In early AD, neurogenic impairment can be observed, accompanied by hyperreactive astrogliosis. During the treatment of AD, neurogenesis should be promoted, while neuroinflammation should be suppressed. S1R plays a role in both processes. In our experiments, we established a model of early AD induced by Aβ_1–42_, in which acute neuroinflammation, impaired neurogenesis and elevated S1R levels were detected. In this model, two S1R agonists were tested. DMT, binding moderately to S1R but with a high affinity to 5-HT receptors, negatively influenced neurogenesis in the Aβ_1–42_-induced rodent model, probably explained by its acting on the latter receptor class. In contrast, the highly selective S1R agonist, PRE084 improved the proliferation and differentiation of hippocampal stem cells, manifesting in a quantitative increase in progenitor cells and immature neurons. Further experiments are required to investigate the main molecular pathways targeted by DMT, through which it affects neurogenesis and the survival of mature neurons. Moreover, DMT and PRE084 were found to significantly reduce Aβ_1–42_-induced hyperreactive astrogliosis. However, none of these ligands had a remarkable effect on microglial activation. Therefore, further studies are needed to clarify the role of DMT and PRE084 in neuroinflammatory processes induced by Aβ_1–42_, resembling the changes characteristic of AD.

## Data Availability

Not applicable.

## Supplementary Materials

The following supporting information can be downloaded at: https://www.mdpi.com/article/10.3390/ijms23052514/s1.

## Abbreviations

5-HT	5-hydroxytryptamine
AD	Alzheimer’s disease
APP	amyloid precursor protein
Aβ	amyloid-β
BrdU	5-Bromo-2′-Deoxyuridine
BSA	bovine serum albumin
CNS	central nervous system
DCX	doublecortin
DG	dentate gyrus
DMT	N,N-dimethyltryptamine
ER	endoplasmic reticulum
GAPDH	glyceraldehyde 3-phosphate dehydrogenase
GFAP	glial fibrillary acidic protein
HC	hippocampus
Iba1	ionized calcium-binding adapter molecule 1
ICV	intracerebroventricular
IP	intraperitoneal injection
MAM	mitochondria-associated ER membrane
NeuN	neuronal nuclei
NMDA	N-methyl-d-aspartate receptor
NSC	neuronal stem cell
PFA	paraformaldehyde
PRE084	2-(4-morpholinethyl)-1-phenylcyclohexanecarboxylate
SR	sigma receptor
S1R	sigma-1 receptor
SGZ	subgranular zone
UPR	unfolded protein response
WB	western blot analysis
