# Supplementary material for: Impact of Two Neuronal Sigma-1 Receptor Modulators, PRE084 and DMT, on Neurogenesis and Neuroinflammation in an Aβ1–42-Injected, Wild-Type Mouse Model of AD
Source: Int J Mol Sci. 2022 Feb 24;23(5):2514. doi: 10.3390/ijms23052514 (PMC8910266; doi:10.3390/ijms23052514)
Supplement: Supplementary file 1 [file ijms-23-02514-s001.zip › ijms-1542352-supplementary.pdf]

Supplement

Western blot (WB) experiments for the determination of the changes in the levels of two proinflammatory cytokines, IL1β and TNFα

Methods:

The experimental protocol was identical with that described in 5.3. The applied antibodies were the following. For IL1β: anti-IL-1β (clone E7-hIL1β) sc-32294, Santa Cruz Biotechnology, Inc., dilution 1:2000, and for TNFα: anti-TNFα (clone 52B83) sc-52746, Santa Cruz Biotechnology, Inc., dilution 1:5000.

Results:

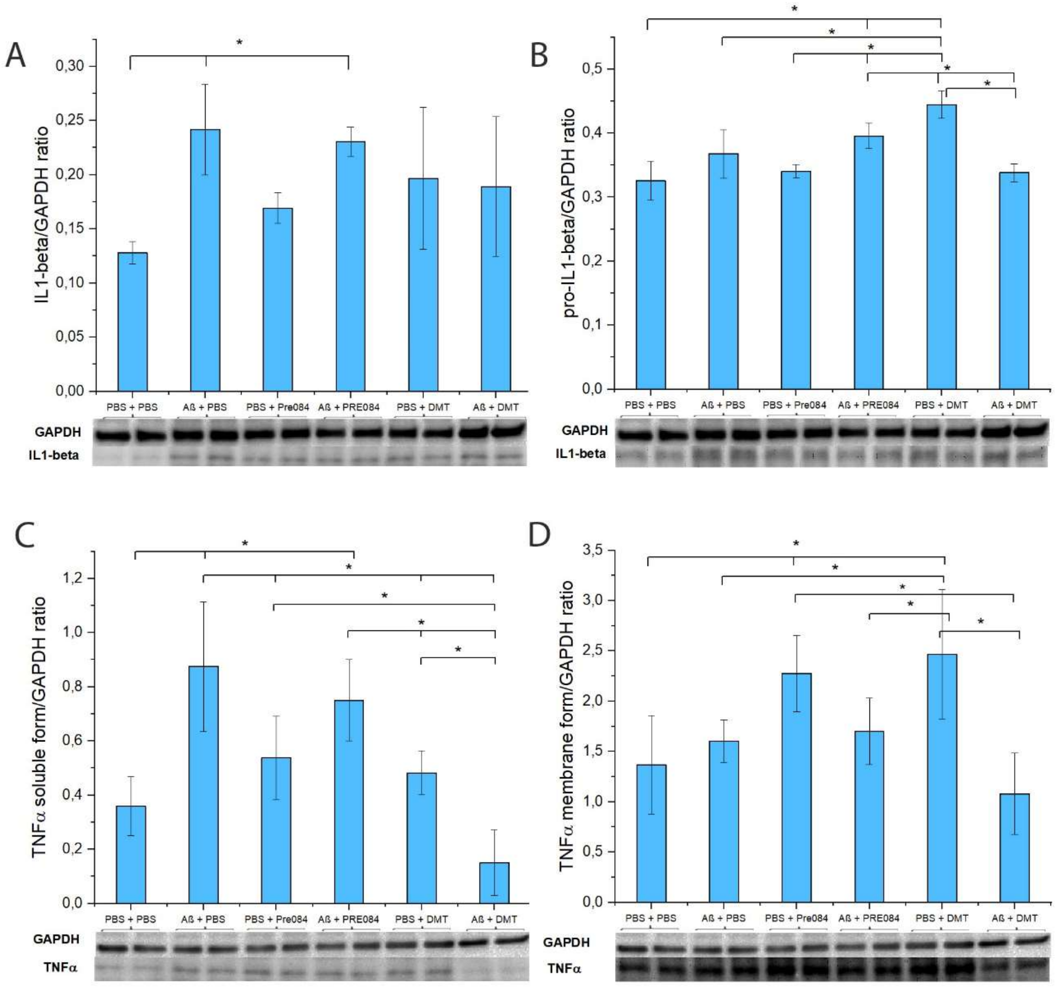

Figure S1. Results for the Western Blot (WB) analyses and representative gel images. Significant differences were detected among the groups (ANOVA: p≤0.0001) in each case. **A.** Analysis of soluble IL1β levels. Compared to PBS-PBS-treated mice, the IL1β protein levels were significantly elevated in the $A\beta_{1-42}$-PBS (p=0.006), and $A\beta_{1-42}$-PRE084 (p=0.012) groups. **B.** Analysis of pro-IL1β levels. Significant differences were observed between the following groups: PBS-PBS *vs.* $A\beta_{1-42}$-PRE084 (p=0.004), PBS-PBS *vs.* PBS-DMT (p≤0.0001), $A\beta_{1-42}$-PBS *vs*. PBS-DMT (p=0.002), PBS-PRE084 *vs*. $A\beta_{1-42}$-PRE084 (p=0.016), PBS-PRE084 *vs*. PBS-DMT (p≤0.0001), $A\beta_{1-42}$-PRE084 *vs*. PBS-DMT (p=0.028), $A\beta_{1-42}$-PRE084 *vs*. $A\beta_{1-42}$-DMT (p=0.013), $A\beta_{1-42}$-DMT *vs*. PBS-DMT (p≤0.0001). **C.** Analysis of soluble TNFα

levels. Significant differences were observed between the following groups: PBS-PBS *vs.* $A\beta_{1-42}$-PBS (p p≤0.0001), PBS-PBS *vs*. $A\beta_{1-42}$-PRE084 (p=0.002), $A\beta_{1-42}$-PBS *vs.* PBS-DMT (p=0.002), $A\beta_{1-42}$-PRE084 *vs.* PBS-DMT (p=0.022), $A\beta_{1-42}$-PBS *vs.* $A\beta_{1-42}$-DMT (p≤0.0001), PBS-PRE084 *vs.* $A\beta_{1-42}$-DMT (p=0.002), $A\beta_{1-42}$-PRE084 *vs.* $A\beta_{1-42}$-DMT (p≤0.0001), $A\beta_{1-42}$-DMT *vs.* PBS-DMT (p=0.006). **D.** Analysis of membrane-bound TNFα levels. Significant differences were observed between the following groups: PBS-PBS *vs*. PBS-PRE084 (p=0.024), PBS-PBS *vs*. PBS-DMT (p=0.009), $A\beta_{1-42}$-PBS *vs.* PBS-DMT (p=0.030), $A\beta_{1-42}$-PRE084 *vs.* PBS-DMT (p=0.050), $A\beta_{1-42}$-DMT *vs.* PBS-PRE084 (p=0.005), PBS-DMT *vs.* $A\beta_{1-42}$-DMT (p=0.002).
